# The effect of federal and state off-label marketing investigations on drug prescribing: The case of olanzapine

**DOI:** 10.1371/journal.pone.0175313

**Published:** 2017-04-07

**Authors:** Bo Wang, David M. Studdert, Ameet Sarpatwari, Jessica M. Franklin, Joan Landon, Aaron S. Kesselheim

**Affiliations:** 1 Department of Medicine, Stanford University School of Medicine, Stanford, California, United States of America; 2 Program On Regulation, Therapeutics, And Law (PORTAL), Division of Pharmacoepidemiology and Pharmacoeconomics, Department of Medicine, Brigham and Women’s Hospital and Harvard Medical School, Boston, Massachusetts, United States of America; 3 Center for Health Policy/Center for Primary Care and Outcomes Research Stanford Medical School & Stanford Law School, Stanford University, Stanford, California, United States of America; 4 Division of Pharmacoepidemiology and Pharmacoeconomics, Department of Medicine, Brigham and Women’s Hospital and Harvard Medical School, Boston, Massachusetts, United States of America; York University, CANADA

## Abstract

In the past decade, the federal government has frequently investigated and prosecuted pharmaceutical manufacturers for illegal promotion of drugs for indications not approved by the Food and Drug Administration (FDA) (“off-label” uses). State governments can choose to coordinate with the federal investigation, or pursue their own independent state investigations. One of the largest-ever off-label prosecutions relates to the atypical antipsychotic drug olanzapine (Zyprexa). In a series of settlements between 2008 and 2010, Eli Lilly paid $1.4 billion to the federal government and over $290 million to state governments. We examined the effect of these settlements on off-label prescribing of this medication, taking advantage of geographical differences in states’ involvement in the investigations and the timing of the settlements. However, we did not find a reduction in off-label prescribing; rather, there were no prescribing changes among states that joined the federal investigation, those that pursued independent state investigations, and states that pursued no investigations at all. Since the settlements of state investigations of off-label prescribing do not appear to significantly impact prescribing rates, policymakers should consider alternate ways of reducing the prevalence of non-evidence-based off-label use to complement their ongoing investigations.

## Introduction

After a new drug is initially approved by the Food and Drug Administration (FDA), it can be prescribed for any purpose, including non-FDA-approved, “off-label” uses. However, federal and state laws restrict marketing for off-label uses. Manufacturers are prohibited from initiating discussions about off-label uses in marketing materials (although manufacturers are permitted to distribute pertinent published clinical studies to health care providers in a non-promotional manner) [1]. The goal of these rules is to strike an appropriate balance between distributing useful scientific information to the medical community and protecting patients from the risks of using therapeutics for non-evidence-based indications.

Despite these rules, off-label prescribing is highly prevalent, ranging from 30 percent for oncology drugs to over 70 percent for atypical antipsychotic medications in children [2, 3]. While some drugs may be backed by sufficient evidence for use in clinical situations not approved by the FDA, most are not [4]. Furthermore, off-label prescribing not only adds substantially to health care costs, but is also a threat to public health; for example, elderly patients with dementia treated with atypical antipsychotics like olanzapine (Zyprexa) have an increased rate of death [5].

In the past decade, pharmaceutical manufacturers have frequently been investigated at the federal and state level for engaging in off-label promotion [6, 7, 8, 9]. In a previous study, we found that the federal government’s multi-billion dollar settlement with Warner-Lambert (later acquired by Pfizer) for illegal off-label promotion of the anti-seizure medication gabapentin (Neurontin) was associated with a decrease nationwide in off-label prescribing of the drug [10]. However, it was difficult to make a causal connection between the enforcement action and the change in prescribing in that study, because of a switch of the drug to generic suppliers around the time of settlement.

To extend and deepen our understanding of the effect of large off-label marketing settlements on prescribing practices, we turned to examine legal action over another drug: olanzapine. On January 15, 2009, the US Department of Justice (DOJ) announced that Eli Lilly had agreed to pay over $1.4 billion for illegally promoting olanzapine. The settlement followed an investigation of the company for promoting such non-FDA-approved uses as treatment of dementia, aggression, and generalized sleep disorder [11]. In addition, between 2008 and 2010, Lilly paid over $290 million to settle a series of separate state investigations relating to illegal off-label promotion of olanzapine.

Because olanzapine involved federal and state investigations that settled at different times, it presented a case study for exploring the relationship between legal prosecution and off-label prescribing. We hypothesized that federal settlements against pharmaceutical manufacturers for off-label marketing may be interpreted differently by the medical community in states that prosecuted, owing to increased visibility and awareness of the settlement results. We further hypothesized that such differences would manifest in prescribing outcomes, with more aggressive prosecution of off-label promotion of olanzapine being associated with decreases in the rate of off-label prescription of this drug.

## Methods

This study was approved by the Brigham and Women’s Hospital Institutional Review Board. All participants provided informed consent to have their information stored in the Optum Research database, the data source for our study. No informed consent was obtained for the purposes of this study because the data was analyzed anonymously.

### Regulatory background

Violations of off-label marketing regulations are often brought to the attention of the DOJ by whistleblowers, many of whom are former company employees with inside information about these practices [12]. The DOJ must then decide whether to launch a full investigation of the alleged infractions and whether to join lawsuits against the drug manufacturers filed by the whistleblowers themselves under the False Claims Act (FCA). The FCA allows for up to triple damages against entities that willfully submit false claims for reimbursement to the government—in this case, the prescriptions induced by illegal off-label promotion, which are paid for by Medicare, Medicaid, or other government insurers [13]. Since 2004, civil and criminal fines totaling over $15 billion have been levied against nearly all of the major drug manufacturers for FCA violations relating to illegal off-label promotion of their products [6, 7, 8, 9].

State governments also pay for drugs in a variety of ways, including as co-funders of Medicaid programs. They are permitted to join federal investigations, or to pursue their own enforcement actions under state law. Some states have enacted their own versions of the FCA, and all states have consumer protection statutes that may provide grounds for prosecuting the promotion of off-label prescribing. In the case of olanzapine, 32 states and the District of Columbia joined the federal investigation, 13 states pursued separate state-level investigations against Lilly, 1 state joined the federal investigation and pursued its own, and 6 states did neither (Table 1) [14, 15].

**Table 1:** State participation and settlements in illegal off-label marketing investigations of Lilly for olanzapine.

Action	Participating States	Settlement(s)
Joined federal False Claims Act investigation	Alabama, Arizona, California, Delaware, Florida, Hawaii, Illinois, Indiana, Iowa, Kansas, Maine, Maryland, Massachusetts, Michigan, Missouri, Nebraska, Nevada, New Jersey, New York, North Carolina, North Dakota, Ohio, Oklahoma, Oregon, Pennsylvania, Rhode Island, South Dakota, Tennessee, Texas, Vermont, Washington, Wisconsin, District of Columbia	10/2008: $62 million^1^1/2009: $1.4 billion
Initiated state-level investigation	Alaska, Arkansas, Connecticut, Idaho, Louisiana, Minnesota^2^, Mississippi, Montana, New Mexico, Pennsylvania^2^^#^, South Carolina, Utah, West Virginia	3/2008: AK—$15 million 8/2009: WV—$22.5 million 9/2009: CT—$25.1 million 10/2009: ID—$13 million 10/2009: SC—$45 million 11/2009: UT—$24 million 12/2009: NM—$15.5 million 2/2010: MS—$18.5 million 2/2010: AR—$18.5 million 2/2010: MT—$13 million 4/2010: LA—$20 million
No investigation	Colorado, Georgia, Kentucky, New Hampshire, Virginia, Wyoming	No settlements

^1^Settlement awarded under consumer protection laws of these states/district

^2^No settlement as of December 2015

^#^Pennsylvania joined the federal False Claims Act investigation and also launched a separate state investigation

### Approvals of olanzapine

The FDA initially approved the marketing of olanzapine in September 1996 for the treatment of psychotic disorders in adults [16]. It was approved for certain types of adult bipolar disorder in March 2000, followed by formal approval for treatment of schizophrenia in November 2000 [17, 18]. In December 2009, olanzapine was approved for treatment of schizophrenia and certain types of bipolar disorder in adolescents (13 to 17 years old) [19].

Thus, for the study period (2004–2011), there were no changes to the approved uses of olanzapine in adults, but several changes to approved uses in adolescents. Consequently, we chose to confine our analysis to prescriptions written for adult patients.

### Timeline of federal and state actions

We used the Public Access to Court Electronic Records database [20], press releases from the DOJ and other sources [21], and archives of US federal court filings to identify the timeline of federal- and state-level FCA investigations related to olanzapine.

Table 1 shows the states that joined or launched investigations, and the related settlements. The federal investigation was filed in February 2003 [22]. It charged Lilly with willfully engaging in off-label promotion of olanzapine between September 1999 and 2005. Thirty-two states and the District of Columbia eventually joined this investigation. The investigation concluded in January 2009 with Lilly agreeing to pay a global fine of $1.415 billion ($800 million civil settlement, $515 million criminal fine, and $100 million asset forfeiture) and enter into a Corporate Integrity Agreement aimed at preventing similar violations. In October 2008, Lilly had also agreed to pay $62 million in October 2008 to settle consumer protection lawsuits brought by the same 32 states [11].

In addition to the federal investigation, independent state-level investigations were filed by Pennsylvania (a participant in the federal investigation) and 12 other states that did not join the federal investigation: Alaska, West Virginia, Connecticut, Idaho, South Carolina, Utah, New Mexico, Mississippi, Arkansas, Montana, Louisiana, and Minnesota. All of these states, except for Pennsylvania and Minnesota, reached settlements with Lilly between March 2008 and April 2010, resulting in fines ranging from $13 million to $45 million. In total, 44 states were involved in either federal and/or state investigations and six states were involved in neither: Wyoming, Colorado, Kentucky, Georgia, Virginia, and New Hampshire.

### Data

Our study dataset was extracted from the Optum Research Database from 2004–2011, which contains medical and pharmacy data on insurance claims for more than 14 million beneficiaries of UnitedHealth’s commercially insured population and which has been utilized in previous studies [23, 24]. The population covered by this database reflects the UnitedHealth’s nationwide reach, with demographics similar to the age distribution of the US census for both sex and age groups <65 years (data from Georgia were excluded due to validation concerns).

### Study sample

We identified olanzapine prescription claims in the Optum Research Database relating to adult patients (≥18 years of age) and aggregated them by calendar month. The outcome of interest in our analyses was the monthly incidence of new adult users of olanzapine. A “new user” was defined as a patient prescribed olanzapine who had had no previous fills for this medication in the previous 180 days. Only new users who maintained continuous insurance eligibility, defined as ≥1 inpatient or outpatient claim and ≥1 filled prescription of any medications during the prior 180 days, were included to ensure that that we did not include patients who may have filled prescriptions for olanzapine with prior insurers. Enrollees could be counted as new users more than once if more than 180 days elapsed since their last medication fill.

### Identification of prescriptions for on-label and off-label indications

We determined whether the new olanzapine users in the sample were prescribed the drug for an on-label or off-label indication by reference to other information in the Optum Research Database pertaining to the users’ medical conditions. Specifically, new users with on-label indications were patients who, within the 180 days prior to the prescription, had diagnosis codes that matched the FDA-approved indications for use of olanzapine (*i*.*e*., schizophrenia and/or bipolar disorder) or had concurrent drug prescriptions related to one or more of these diagnoses (see S1 Appendix for ICD-9-CM codes and list of concurrent drugs). All other new users were classified as patients who received the olanzapine prescription for an off-label indication.

### Analysis

To estimate whether the legal settlements affected prescription patterns, we conducted interrupted time series analyses. The outcome variable in these analyses was the number of new users of olanzapine per 100,000 active adult enrollees per month. New users were divided into those who were prescribed olanzapine for an off-label indication and those who were prescribed olanzapine for an on-label indication.

We began by examining the effect of the January 2009 federal settlement. Our focus was on possible effects within states that were part of the federal investigation, but for comparative purposes we also examined trends before and after the federal settlement in the other two groups of states (those that launched independent state investigations and those that were not involved in any investigations). The interval for this analysis was July 2004 through January 2011, consisting of a pre-settlement period running from July 2004 to December 2008, a breakpoint month in January 2009 during which the federal settlement occurred, and a post-settlement period running from February 2009 to January 2011. The time series for new olanzapine prescription tracking commenced on July 2004 because this was the earliest month of complete data available to us and was early enough to permit tracking of new users of olanzapine through most of the federal investigation. Because it participated in both federal and independent state investigations, Pennsylvania was excluded from the analysis.

Next, we examined effects of settlements on new olanzapine use in 11 states that had pursued their own state-level investigation. Minnesota was excluded because it had not reached a settlement as of December 2015; Pennsylvania was excluded because it had not reached a state settlement by this time and because it was also part of the federal investigation. Since state settlements were reached on different dates between March 2008 and April 2010, the breakpoints in the analysis were state specific, relating to the actual month of settlement in each state. The times series consisted of 24 months before and after each breakpoint month (49 months in total).

For each interrupted time series, we fit a Poisson model, using generalized estimating equations and an AR(1) correlation model. The number of enrollees was included as an offset. We allowed for separate trends in each group, before and after the interruption. Difference in differences was performed for the federal investigation analysis to test for variations in incidence of olanzapine users following federal settlement among the three groups of states; the same technique was also performed for the state investigations analysis to evaluate the incidence of olanzapine users following state settlements for off-label compared to on-label use. All analyses were performed using Stata 13.1 (StataCorp LP, College Station, TX).

## Results

There were 25,471 new olanzapine users in our study sample. A total of 10,457 of these new users were prescribed the drug for off-label indications and 15,014 were prescribed the drug for on-label (i.e. approved) indications.

### Prescription trends following federal settlement

**Fig 1:**
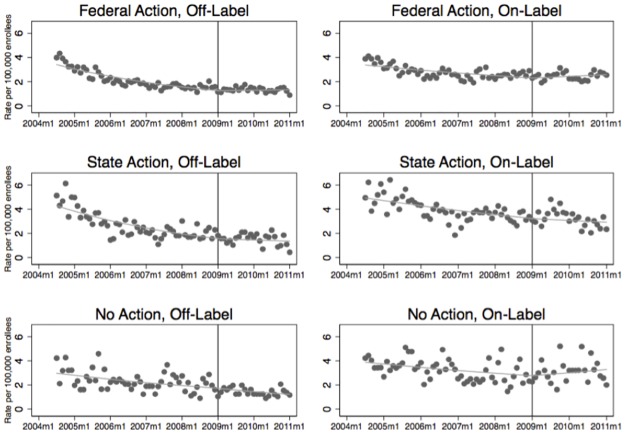
Time-series analysis of new adult users of olanzapine for on- and off-label indications before and after federal settlement under the False Claims Act, with states grouped by type of investigation pursued against the manufacturer, July 2004 through January 2011. The first time interval included the 54 months from July 2004 through December 2008. The US Department of Justice announced the federal False Claims Act settlement with Lilly in January 2009, which served as the breakpoint for our analysis. The second time period spanned the 24 months from February 2009 through January 2011.

Among states joining the federal investigation, there were 24,429 new olanzapine users between July 2004 and January 2011, consisting of 10,049 patients receiving new prescriptions for off-label indications and 14,380 for approved indications. Fig 1 shows trends for overall olanzapine prescriptions in the three groups of states—those that joined federal investigation, those that pursued independent state investigation, and those that pursued neither—before and after the federal settlement was announced in January 2009. The vertical lines in the plots indicate the date of the federal settlement. The plots suggest a decreasing rate of new off- and on-label users in all three groups of states, at least in the pre-settlement period. No clear changes in this trend were evident in the post-settlement period, even among states that were part of the federal settlement.

The regression analyses largely confirm these results (Table 2). The monthly rate of new off-label users of olanzapine was decreasing in all three groups of states in the period leading up to federal settlement (trend coefficient = 0.982 for federal action, 0.981 for independent state action, and 0.990 for no action; trend coefficient of 1 signifies no utilization change). After the settlement, there was a very small increase in the rate of new off-label users in states that were part of the federal investigation (trend coefficient 1.002, p<0.001). States that pursued their own investigation and states that had no investigation continued to experience decreases in the incidence of new off-label users of olanzapine, but the size of these decreases did not change after the federal settlement (trend coefficient = 0.994, p = 0.10 for independent state action; trend coefficient = 0.986, p = 0.71 for no action).

**Table 2:** Statistical analyses of utilization trends of olanzapine before and after federal False Claims Act settlement, using Poisson regression models, July 2004 to January 2011.

Off-Label, federal investigation				
	Before Federal Settlement	After Federal Settlement	P-Value for Change	P-Value for Comparing Changes among State Groups
Federal investigation	0.982	1.002	<0.0001	0.0004
State investigation	0.981	0.994	0.10	
No investigation	0.990	0.986	0.71	
On-Label, federal investigation				
	Before Federal Settlement	After Federal Settlement	P-Value for Change	P-Value for Comparing Changes among State Groups
Federal investigation	0.993	1.005	<0.0001	0.001
State investigation	0.992	0.996	0.52	
No investigation	0.994	1.007	0.09	

Breakpoint designated as the January 2009, the month of federal False Claims Act settlement. The numbers in the “before” and “after” columns are incidence rate ratios, analogous to the slopes in a linear regression. Trend coefficient of 1 signifies no change in utilization; trend coefficient indicates increase in utilization if >1 and decrease in utilization if <1.

The trends in on-label use were similar, with states that were part of the federal investigation experiencing a small but significant increase in the incidence of new on-label users in the post-settlement period (pre-settlement coefficient = 0.993, post-settlement coefficient = 1.005, p<0.0001). States pursuing their own investigations had non-significant declines in new on-label users after the federal settlement while those filing no investigation experienced a non-significant increase in trend (pre-settlement coefficient = 0.992, post-settlement coefficient = 0.996, p = 0.52 for state action; pre-settlement coefficient = 0.994, post-settlement coefficient = 1.007, p = 0.09 for no action)

### Trends following independent state settlements

Among states that pursued their own independent investigations and did not join the federal investigation, there were 1,042 new users during the study period. These new users consisted of 408 patients who received prescriptions for off-label indications and 634 patients who received prescriptions for on-label indications.

**Fig 2:**
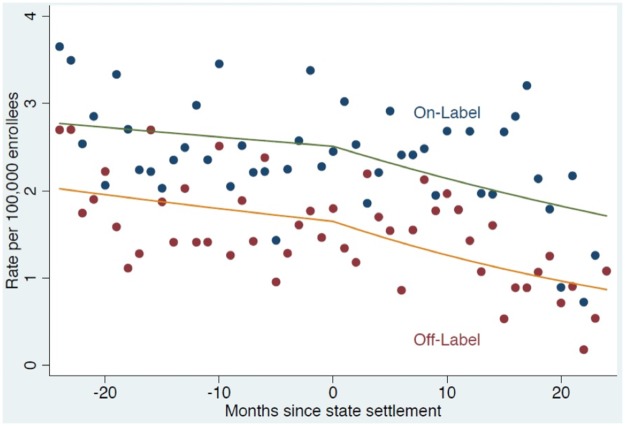
Time-series analysis of new adult users of olanzapine for on- and off-label indications before and after state off-label settlements. The 11 states that pursued and successfully settled independent state-level investigations were included in this analysis: Alaska, West Virginia, Connecticut, Idaho, South Carolina, Utah, New Mexico, Mississippi, Arkansas, Montana, Louisiana (Minnesota and Pennsylvania also pursued state investigations but had not yet reached a settlement at the time of this paper’s final submission). The figure shows aggregated incident utilization data for olanzapine among these 11 states, with the first time interval spanning the two years before each state’s settlement. The month of each state’s settlement served as the breakpoint for our analysis. The second time period spanned the two-year period following the state settlements.

Both on-label and off-label prescribing in these states decreased during pre-settlement periods (trend coefficient = 0.992 for off-label, 0.996 for on-label), and declined more steeply after the settlements (trend coefficient = 0.974 for off-label, 0.984 for on-label), however the difference in the trend lines was not statistically significant (p = 0.24 for off-label, p = 0.33 for on-label; see Fig 2 and Table 3).

**Table 3:** Statistical analyses of utilization trends of olanzapine before and after state off-investigation settlements, using Poisson regression models.

	Before State Settlement	After State Settlement	P-Value for Change	P-Value for Difference in Changes
Off-Label	0.992	0.974	0.24	0.74
On-Label	0.996	0.984	0.33	

Breakpoint designated as the month of the state investigation settlements (from March 2008 for Alaska to April 2010 for Louisiana). The numbers in the “before” and “after” columns are incidence rate ratios, analogous to the slopes in a linear regression. Trend coefficient of 1 signifies no change in utilization; trend coefficient indicates increase in utilization if >1 and decrease in utilization if <1.

## Discussion

This study detected no major reductions in the incidence of off-label prescribing of olanzapine following a large federal settlement against the manufacturer in 2009, or following a series of individual state settlements concluded between 2008 and 2010. Moreover, there was little difference between off-label and on-label prescribing trends during the study period. The incidence of new olanzapine users was decreasing across the board. The only significant post-settlement change we observed was counter-intuitive: the federal settlement was associated with a small but significant increase in off-label prescribing in states that were party to the settlement.

The decreasing rate of olanzapine prescription we observed during the pre-settlement period may be attributable to emerging evidence during this time of metabolic, cerebrovascular, and mortality risks associated with antipsychotic use, which culminated in an official warning from the FDA in 2005 regarding adverse effects from the atypical antipsychotics in patients with dementia-related psychosis [25, 26, 27].

The lack of an association between settlements and off-label prescribing is more difficult to explain. One possibility is that off-label prescribing of olanzapine did not decline post-settlement because the manufacturer’s marketing practices persisted. The federal penalties imposed on Lilly in 2009 were a fraction of the $4.7 to $5.0 billion in annual revenues the company earned from the drug between 2008–2010, the years encompassing the federal and state settlements [28, 29], and may have been insufficient to deter illicit marketing. The financial penalties in state settlements, which ranged from $13 million to $45 million, were even more anemic relative to sales revenue; these are sums Lilly would have recouped in a few days of olanzapine sales.

In addition, the FDA issued a guidance document in early 2009 allowing drug and device companies to distribute reprints of medical journal articles relating to off-label uses of their products, without regard to the robustness of the study or the journal’s reputation [30]. This move would have created opportunities for manufacturers to continue promoting unapproved uses of drugs such as olanzapine by circulating carefully-culled published studies drawing favorable conclusions. Thus, prescribers may have continued to have been influenced by ongoing illegal and legal forms of off-label promotion during the post-settlement periods.

A second possibility is that the die was already cast. If the manufacturer’s earlier off-label promotion efforts had already shaped physicians prescribing practices around olanzapine in enduring ways, the settlement and the Corporate Integrity Agreement with Lilly aimed at guarding against further improper marketing may have had little effect in practical terms.

Our study has limitations. The dataset consisted of patients from one commercial insurer and the physicians who treated them, and they may not be representative of the wider population of olanzapine users and prescribers. We did not adjust for certain factors that may have affected prescribing trends such as media coverage of Lilly’s off-label promotion and safety problems associated with this medication that the manufacturer had previously downplayed; the emergence of such factors may have contributed to the decline observed in prescribing patterns [31, 32, 33]. In addition, the number of new olanzapine users among states pursuing independent investigations was only a fraction of new users in the federal investigation analysis, and may be limited in power to detect differences in prescribing patterns following the state settlements. Most importantly, our case study examined a single drug. We cannot rule out the possibility that off-label investigations and settlements have different effects on prescription practices associated with other drugs.

## Conclusion

Physicians should be encouraged to make independent, evidence-based decisions regarding the appropriate therapies to prescribe for their patients, both for FDA-approved and off-label purposes. To optimize prescribing decisions, physicians must have access to medical information that is balanced, accurately emphasizing benefits and harms, such as those provided by the International Society of Drug Bulletins and Prescrire International [34, 35]. Unfortunately, such a model is at odds with the goals of pharmaceutical promotion, which is primarily intended to generate revenue. For this reason, despite the fact that certain off-label uses may be justified and beneficial for some patients, off-label promotion has traditionally been restricted. The restrictions are intended to improve the quality of information transmitted to prescribers, reduce unsafe drug use, and encourage manufacturers to conduct clinical studies to investigate promising off-label indications and submit the data for formal FDA review and authorization.

Violation of these rules by manufacturers has led to government investigations and substantial settlements. However, this study of olanzapine joins our prior study of gabapentin (Neurontin) in finding no clear impact of litigation—either during the investigation phase or following settlements—on prescribing of the drugs at issue.^11^ The message is sobering. Enforcement actions may still be socially valuable: they hold violators to account (at least nominally), signal societal disapproval, and raise revenue for the government. Furthermore, it is conceivable that the actions may have a general deterrent value by dissuading improper marketing practices in the industry generally and in the long run. However, our findings raise doubts about the potency of federal and state prosecutions in altering the off-label prescription patterns that are most closely related to the allegedly illegal marketing practices.

One conclusion to draw from this result is that regulators must up the disincentives, financial and otherwise, that manufacturers and their corporate officers face if they engage in off-label marketing, either through sales agents or key opinion leaders. Another conclusion is that the prosecutorial approach and the deterrence rationale behind it are inherently limited. In recent years, other efforts have been aimed at improving the quality of evidence surrounding off-label indications, including in France in 2012 with the “Temporary Recommendations for Use” (RTUs), which implemented a framework to more closely gather and monitor data for off-label prescribing of medications during the immediate post-approval years. However, only a small fraction of patients receiving RTU-designated drugs for off-label purposes were ever registered [36, 37]. Other strategies for stemming the problem of inappropriate prescribing—such as government investment in unbiased educational opportunities for physicians to learn about evidence-based prescribing—should not be forgotten.

## Supporting information

S1 Appendix ICD-9 diagnostic codes and concurrent prescription drugs. X = any digit.
(DOCX)
