# Supplementary material for: The effect of federal and state off-label marketing investigations on drug prescribing: The case of olanzapine
Source: PLoS One. 2017 Apr 7;12(4):e0175313. doi: 10.1371/journal.pone.0175313 (PMC5384770; doi:10.1371/journal.pone.0175313)
Supplement: S1 Appendix — X = any digit. (DOCX) [file pone.0175313.s001.docx]

**1A. PSYCHOTIC DISORDER**

290.8x other specified senile psychotic conditions

290.9x unspecified senile psychotic condition

295.xx schizophrenia

297.xx paranoid states

298.xx other nonorganic psychoses

299.xx psychoses with origin specific to childhood

780.1x hallucinations

**2. BIPOLAR DISORDER**

296.0x manic disorder, single episode

296.1x manic disorder, recurrent episode

296.4x bipolar affective disorder, manic

296.5x bipolar affective disorder, depressed

296.6x bipolar affective disorder, mixed

296.7x bipolar affective disorder, unspecified

296.8x bipolar affective disorder, other and unspecified

296.99 other specified affective psychoses (e.g., mood swings)

**3. CONCURRENT DRUG PRESCRIPTIONS**

Chlorpromazine, trifluoperazine, clozapine, lurasidone, loxapine, asenapine, ziprasidone, risperidone, paliperidone, quetiapine, aripiprazole, iloperidone, molindone, thioridazine, mesoridazine, reserpine, haloperidol, lamotrigine, fluoxetine/olanzapine, lithium, divalproex sodium, valproic acid
